# Temporal turnover of viral biodiversity and functional potential in intertidal wetlands

**DOI:** 10.1038/s41522-024-00522-8

**Published:** 2024-06-19

**Authors:** Mengzhi Ji, Yan Li, Jiayin Zhou, Wen Song, Yuqi Zhou, Kai Ma, Mengqi Wang, Xia Liu, Yueyue Li, Xiaofan Gong, Qichao Tu

**Affiliations:** 1 Institute of Marine Science and Technology, Shandong University, Qingdao, Shandong Province China; 2 Southern Marine Science and Engineering Guangdong Laboratory (Zhuhai), Guangzhou, China; 3 Present Address: Institute of Soil and Water Resources and Environmental Science, College of Environmental and Resource Sciences, Zhejiang University, Hangzhou, China

**Keywords:** Environmental microbiology, Metagenomics

## Abstract

As the central members of the microbiome networks, viruses regulate the composition of microbial communities and drive the nutrient cycles of ecosystems by lysing host cells. Therefore, uncovering the dynamic patterns and the underlying ecological mechanisms mediating the tiniest viral communities across space and through time in natural ecosystems is of crucial importance for better understanding the complex microbial world. Here, the temporal dynamics of intertidal viral communities were investigated via a time-series sampling effort. A total of 1911 viral operational taxonomic units were recovered from 36 bimonthly collected shotgun metagenomes. Functionally important auxiliary metabolic genes involved in carbohydrate, sulfur, and phosphorus metabolism were detected, some of which (e.g., *cysH* gene) were stably present within viral genomes over time. Over the sampling period, strong and comparable temporal turnovers were observed for intertidal viromes and their host microbes. Winter was determined as the pivotal point for the shifts in viral diversity patterns. Notably, the viral micro-diversity covaried with the macro-diversity, following similar temporal patterns. The relative abundances of viral taxa also covaried with their host prokaryotes. Meanwhile, the virus–host relationships at the whole community level were relatively stable. Further statistical analyses demonstrated that the dynamic patterns of viral communities were highly deterministic, for which temperature was the major driver. This study provided valuable mechanistic insights into the temporal turnover of viral communities in complex ecosystems such as intertidal wetlands.

## Introduction

As the most abundant and diverse biological entities, viruses and their host microbes occupy almost all the possible ecological niches in the Earth’s biosphere^1^. Via replication and propagation, viruses play important roles in microbial host metabolism, mortality, and nutrient recycling^2,3^. Critically, the viral infection and lysis of microbial hosts have immediate impacts on microbial taxonomic diversity and can further influence their ecological processes^4^. Viruses can also act as delivery agents for horizontal gene transfer, spreading auxiliary metabolic genes (AMGs) and antibiotic resistance genes, thus regulating the functional traits of their microbial hosts^5,6^. Furthermore, different viral life strategies, such as “Kill-the-Winner” and “Piggyback-the-Winner” models, can result in diverse virus–host interactions, thereby influencing the sustainability and stability of ecosystems^7,8^.

Evaluating the taxonomic diversity and ecological mechanisms of viral communities in complex environments has been challenging for a long period, primarily due to the lack of universally conserved marker genes within viral genomes, such as the prokaryotic 16S rRNA genes^9^. More recently, the advances in high-throughput sequencing and bioinformatics tools have greatly facilitated the development of the nascent field of viral ecology, allowing viral studies to span from the individual to the community level^10,11^. As a result, the taxonomic diversity^12,13^ and functional potential^14,15^ of viruses in various ecosystems can be systematically investigated. Notably, several studies have directed their attention from examining viral macro-diversity variations to delving into micro-diversity variations, which can provide in-depth information for understanding how genetic drift and environmental selection shape viral communities^16,17^.

Revealing the turnovers of taxonomic groups and functional traits across spatial and temporal scales is a critical issue in microbial ecology^18^. Spatial turnover is defined as the changes in species composition or biodiversity from place to place^19^, while temporal turnover represents the number of species eliminated and replaced per unit time^20^. Leveraged by sampling over large scales^12,13,21^, typical biogeographic patterns, such as distance-decay relationship (DDR), followed by viral taxa and functional potentials, have been demonstrated. However, less attention has been received on the studies of viral temporal turnovers compared to their spatial turnovers, especially in complex natural ecosystems^11^. Exploring the temporal turnover patterns, such as taxa–time relationship (TTR)^22^ and time–decay relationship (TDR)^23^, is beneficial for comprehending the environmental adaptability and ecological mechanisms of viral communities.

Intertidal zones are located at the transition regions between the terrestrial and ocean ecosystems. It is a complex ecosystem subjecting to strong interactions of geological, hydrological, physicochemical, and biological factors, performing vital roles in global biogeochemical cycling processes^24^. Owing to their frequent switching between aerobic and anoxic/suboxic environments, intertidal zones are also considered as one of the most complex ecosystems on the Earth^25^. The high environmental heterogeneity and dynamic conditions foster a diverse set of microorganisms, making intertidal zones a natural dynamic model ecosystem for microbial ecological studies. In this study, a time-series sampling effort of intertidal sediments over a 10-month period was made to investigate the temporal dynamics of viruses and their host microbes, aiming to address the following ecological questions: (i) Do viral communities follow similar temporal diversity patterns as their host microbes? (ii) How do viral macro- and micro-diversity respond to the changing environment over time? (iii) How do different ecological processes modulate the temporal dynamic patterns of viral communities? Our results showed strong and comparable temporal turnovers for both viral communities and their host microbes. Multiple AMGs were persistently detected over time, potentially contributing to viral survival and metabolic processes. This study provided a better understanding of the viral communities in a complex ecosystem within an ecological framework.

## Results

### An overview of recovered intertidal viruses and microbes

In this study, 36 mudflat intertidal sediment samples were collected and subjected to shotgun metagenomic sequencing. These samples were collected from an intertidal zone located in Qingdao, China (36.46°N, 120.75°E), in a time-series manner (every two months) spanning from August 2020 to June 2021 (Supplementary Table 1). Multi-sample assembly was performed for each sampling time point. Different methods were simultaneously employed to recover metagenomic viral contigs (mVCs). As a result, a total of 3238 mVCs (≥10 kb or complete) were obtained. These 3238 mVCs were clustered at 95% average nucleotide identity, which approximately corresponds to the species delineation threshold^26^, generating 1911 viral operation taxonomic units (vOTUs), representing the taxonomic composition of viral communities. Among these, 97 (5.1%) were complete or high-quality genomes, and 135 (7.1%) were medium-quality genomes (Supplementary Table 2).

Taxonomic assignment showed that ~82.4% of the recovered vOTUs (1574 of 1911) were *Caudoviricetes* (Supplementary Table 2). And 505 vOTUs (~26%) were found with >0.5× coverage across all sampling time points. Gene-sharing networks showed that 2106 out of 3238 mVCs (~65%) could be clustered, generating 604 viral clusters at the genus level^27^ (Supplementary Fig. 1a, b). Among these, 562 viral clusters (~93%) were composed of mVCs from different time points, suggesting close temporal linkages among intertidal viruses (Supplementary Fig. 1a, b). Yet, only 42 mVCs (~1.3%) were associated with viruses from RefSeq, forming 13 viral clusters (Supplementary Table 3). This implied the high novelty of the recovered intertidal viruses in this study.

To investigate the host diversity of intertidal viruses, 1462 high- and medium-quality (>50% genome completeness and <10% contamination) metagenome-assembled genomes (MAGs) were also recovered from co-assembled contigs. These MAGs were clustered at 95% average nucleotide identity to generate 627 microbial operational taxonomic units (mOTUs), including 11 archaeal mOTUs and 616 bacterial mOTUs (Supplementary Table 4). Among these, the taxonomic diversity of bacterial mOTUs was mainly represented by *Proteobacteria* (271 out of 616), and the taxonomic diversity of archaeal mOTUs was mainly represented by *Euryarchaeota* (6 out of 11) (Supplementary Table 4).

### Functional potential of intertidal viruses

To investigate the functional potential of intertidal viruses, 82,648 protein-coding genes carried by 3238 mVCs were initially clustered at 80% coverage and 60% identity^12,21^, resulting in 41,363 viral protein clusters (vPCs), representing the functional gene composition of viral communities. A total of 10,328 vPCs were assigned to known orthologous groups by searching against the eggNOG database, of which ~41.1% were functionally unknown (Supplementary Table 5). As expected, house-keeping vPCs with functions such as “replication, recombination and repair (L)”, “cell wall/membrane/envelope biogenesis (M)”, and “transcription (K)”, were found with high relative abundances (Supplementary Fig. 2). We then explored whether viruses encoded important AMGs associated with biogeochemical cycles in intertidal zones. A total of 41 vPCs were determined to be AMGs related to carbohydrate, sulfur, and phosphorus metabolism (Supplementary Table 6). Among these, assimilatory sulfate reduction genes, and genes encoding glycoside hydrolases and glycosyltransferases were the most abundant (Supplementary Fig. 3). Moreover, most of these AMGs could be persistently detected through our sampling months (Supplementary Fig. 3). However, the relative abundances of AMGs were quite low compared to other viral functional genes (Supplementary Figs. 2 and 3), especially those related to replication and transcription.

**Fig. 1:**
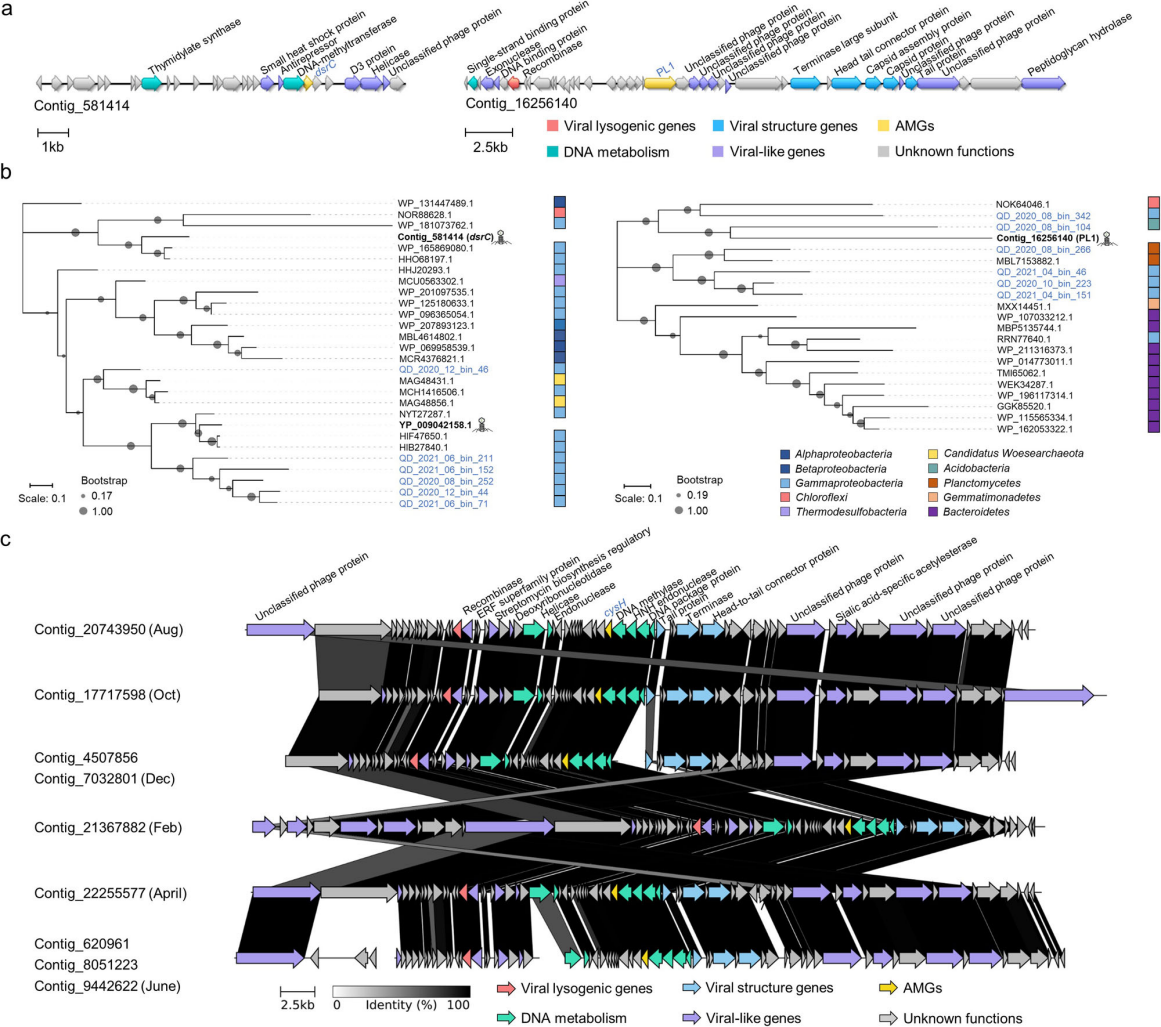
Genomic and phylogenetic analyses of representative viral auxiliary metabolic genes (AMGs). **a** The genomic loci maps of viral contigs carrying *dsrC* and PL1 genes. The arrows represented the locations and directions of the predicted genes in viral contigs. Genes with different functions were marked by different colors. The AMGs were labeled in blue font. **b** Phylogenetic trees of viral *dsrC* and PL1 genes. The phylogenetic trees were constructed based on maximum likelihood algorithms with 500 bootstraps. The AMGs from microbial genomes in public databases were labeled in black font, while those from recovered viral genomes in this current study were labeled in blue font. **c** Comparative genomic analyses of viral contigs carrying *cysH* genes across different time points. The viral contigs assembled from six sampling time points belonged to the same vOTU. The AMGs were labeled in blue font.

Among these AMGs, the dissimilatory sulfate reduction gene (*dsrC*) and the gene encoding polysaccharide lyase (PL1) carried by viruses were found with close homologs in microbial genomes (Fig. 1a, b). Phylogenetic analyses showed that the virus-encoded *dsrC* gene was most similar to the homologs in *Gammaproteobacteria*, suggesting that the AMGs were likely derived from these hosts (Fig. 1b). The virus-encoding PL1 was clustered with microbial PL1 from intertidal MAGs, being most closely related to *Acidobacteria* (Fig. 1b). Interestingly, an assimilatory sulfate reduction gene (*cysH*) was found in mVCs recovered from all sampling months (Fig. 1c). Although these mVCs may have undergone gene deletions and insertions over time, their AMGs may stably persist within viral genomes (Fig. 1c).

### Temporal dynamics of virus–host relationships

**Fig. 2:**
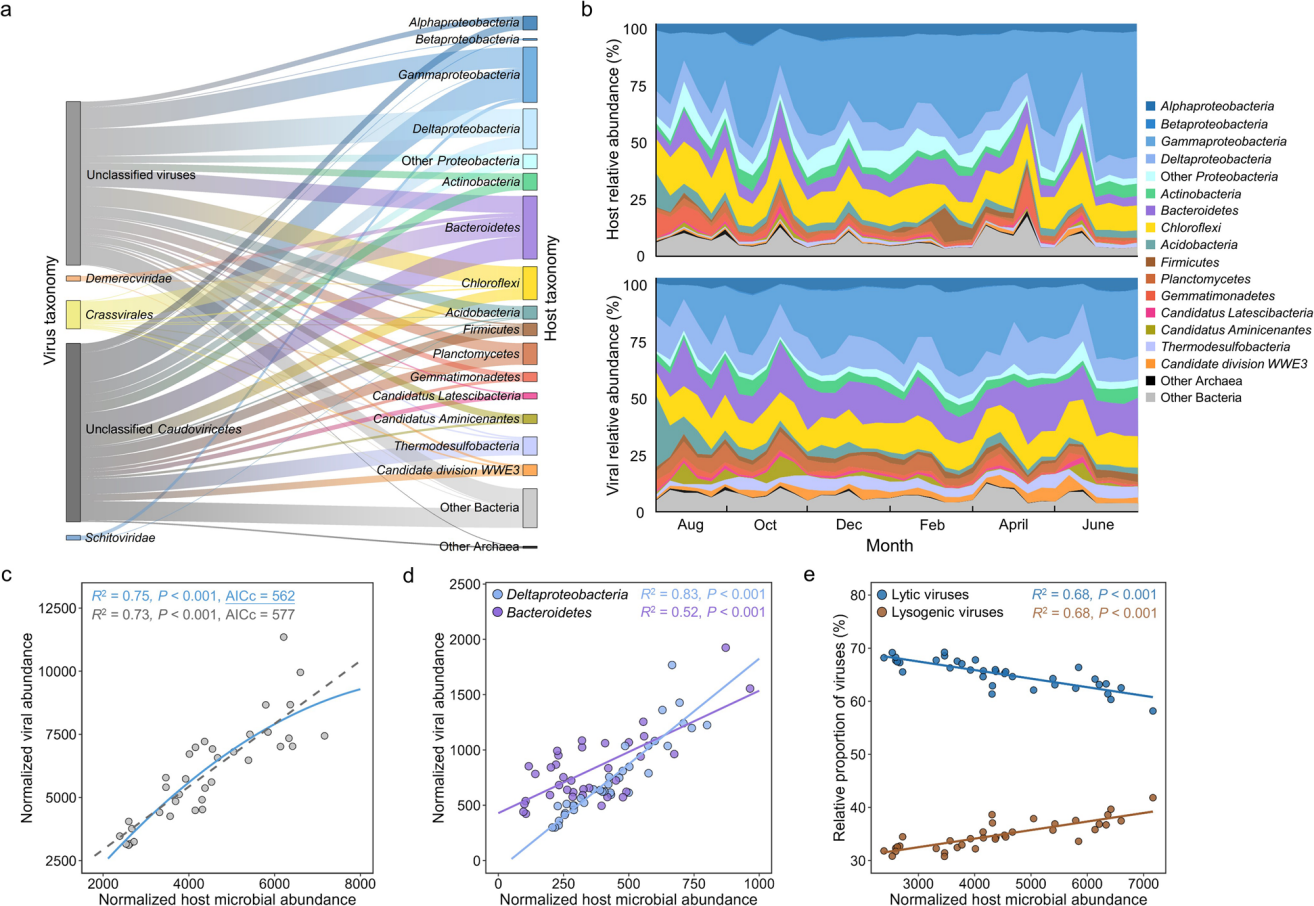
Temporal dynamics of intertidal virus–host relationships. **a** The relationships between viruses and their host microbes. The viruses were grouped at approximately family level, and host microbes were grouped at the phylum level except *Proteobacteria*, which was shown at class level. **b** The relative abundances of viruses and their host microbes grouped by the host lineages from August 2020 to June 2021. **c** The relationship between normalized viral and host abundances. The best polynomial fit (as underlined) was determined based on the corrected Akaike Information Criterion (AICc). **d** The relationships between normalized viral and host abundances for different host lineages. The virus–host relationships for *Deltaproteobacteria* and *Bacteroidetes* are shown in the figure. **e** The relationships between normalized host microbial abundances and the relative proportion of viruses with different lifestyles. For **c**–**e**, Pearson’s correlation coefficients and *P* values of the regressions were presented. All statistical tests were two-tailed.

To infer the potential virus–host relationship, an effort was made to link the recovered 1462 MAGs to the 3238 mVCs via genomic features. As a result, 478 vOTUs and 249 mOTUs were linked to generating virus–host pairs (Supplementary Table 7). The predicted prokaryotic hosts were assigned to 30 phyla. Among these, *Proteobacteria* (163 out of 478) and *Bacteroidetes* (82 out of 478) were the most frequently predicted host phyla (Fig. 2a). The vOTUs that could be assigned with host information mainly belonged to *Caudoviricetes* (Fig. 2a). By linking virus–host taxonomy, viruses belonging to the same order or family were found to have a broad host range (Fig. 2a).

To explore the temporal dynamics of virus–host interactions, six time-series virus–host networks were constructed on the basis of virus–host pairs at the species level. The network size (the number of detected viruses and hosts, *N*) and connectivity (the number of virus–host pairs, *L*) did not clearly change with time, suggesting that the virus–host interactions at the whole community level were relatively stable in the intertidal zone (Supplementary Fig. 4). Dynamic changes in relative abundances of viruses and their host lineages were found over the sampling period (Fig. 2b). Yet, the temporal variation of normalized viral abundances was highly consistent with their prokaryotic hosts (Fig. 2b), suggesting that virus–host interactions potentially regulated the compositional variation of prokaryotic communities. Consequently, stable virus/host abundance ratios (VHRs) were observed for the whole community over the sampling period (Supplementary Fig. 5). For the VHRs of different host lineages, analysis of variance (ANOVA) showed that 10 out of 18 lineages did not differ significantly over time, while the others exhibited relatively strong fluctuations (Supplementary Table 8). Such results suggested that virus–host interactions of different lineages may dramatically vary in response to temporal and seasonal factors.

To investigate the life strategies of intertidal viruses, the correlations between normalized viral and host abundances were also analyzed. The results showed that the normalized viral abundance may not always increase linearly as host microbial abundance increased, leading to a trend of saturation or even decreasing VHRs in intertidal samples with high host microbial abundance (Fig. 2c). When looking at individual microbial taxonomic groups, some host lineages with similar relative abundances exhibited significantly different virus–host abundance relationships (two-way ANOVA, *P* < 0.01), such as *Deltaproteobacteria* and *Bacteroidetes* (Fig. 2d). Also, the VHRs for virus–host pairs belonging to different lineages may harbor opposite trends as the host abundance increased (Supplementary Fig. 6). Strikingly, the relative proportions of lytic and lysogenic viruses exhibited an opposite pattern with host abundance, suggesting that some viruses may choose lysogeny as the life strategy in intertidal samples with high host abundance (Fig. 2e).

### Temporal turnover of viral biodiversity

**Fig. 3:**
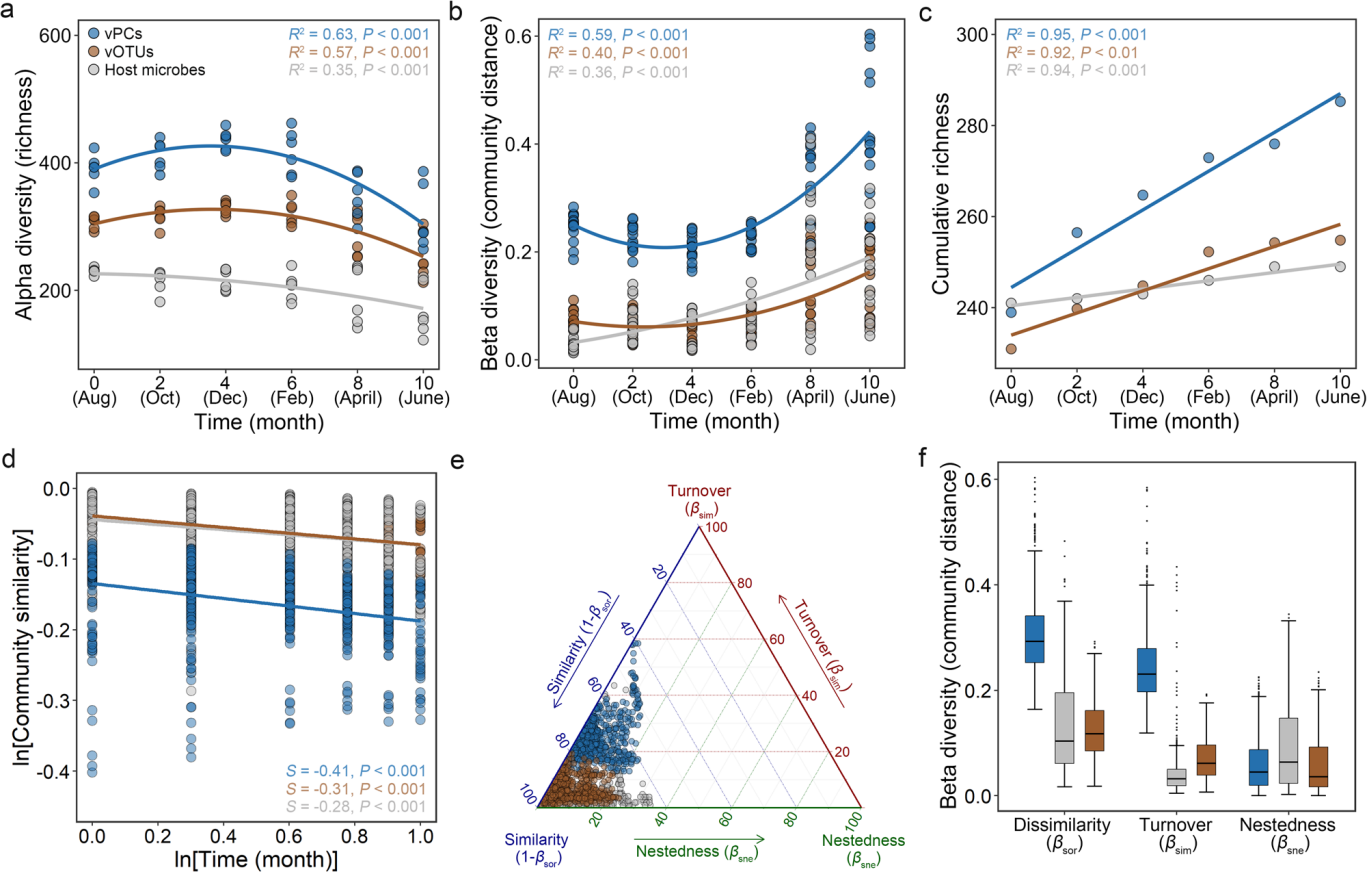
Temporal turnovers of intertidal viruses and their host microbes. **a** The variations in the α-diversity (richness) of viral taxa (vOTUs), viral functional genes (vPCs), and host microbes (mOTUs) along the sampling time. For better visualization, the richness of vOTUs was divided by five, while the richness of vPCs was divided by sixty. **b** The variations in the community distance of viral taxa, viral functional genes, and host microbes along the sampling time. The community distance was measured by Sørensen dissimilarity. **c** Taxa–time relationships describing the relationships of the cumulative richness of viral taxa, viral functional genes, and host microbes with the length of monitored time. **d** Time–decay relationships describing the relationships of decreased community similarity (1-Sørensen dissimilarity) of viral taxa, viral functional genes, and host microbes with the length of monitored time. **e**, **f** The β-diversity (Sørensen dissimilarity) decomposition for viral taxa, viral functional genes, and host microbes. Boxes represented the interquartile range between the first and third quartiles and the median. Whiskers denoted the lowest and highest values within 1.5 times the range of the first and third quartiles. Blank dots represented outlier samples beyond the whiskers. For **a**–**d**, the Pearson’s correlation coefficients and *P* values of the regressions were presented. All statistical tests were two-tailed.

Opposite patterns were observed for α- and β-diversity over the sampling period (Fig. 3a, b). The α-diversity (Richness and Shannon-Wiener indices) of viral taxa (vOTUs) and functional genes (vPCs) exhibited a slight increase from August to December and then rapidly decayed from December to June, while the α-diversity of host microbial communities (mOTUs) decayed continuously (Fig. 3a and Supplementary Fig. 7a). In contrast, the β-diversity (Sørensen and Bray–Curtis dissimilarity) of viral taxa and functional genes slightly decreased from August to December and increased significantly subsequently, while the β-diversity of host microbial communities increased continuously (Fig. 3b and Supplementary Fig. 7b). Moreover, nonmetric multidimensional scaling analyses showed notable differences in the compositions of viral and host microbial communities over different time points (*P* = 0.001, ANOSIM) (Supplementary Fig. 7c).

To examine the temporal turnovers of viral communities, two common patterns in ecology were analyzed, including the TTR and TDR. Of these, TTR describes the increased cumulative taxa richness of a community in a habitat with the length of monitored time^22^, while TDR describes the decreased similarity in community composition with the length of monitored time^23^. As a result, clear TTR and TDR patterns were observed for both viral and host microbial communities (Fig. 3c, d). For TTR, viral communities were found with stronger temporal scaling patterns than host microbial communities (Fig. 3c). For TDR, similar temporal compositional turnover rates were observed for viruses and host microbes (Fig. 3d). The β-diversity decomposition showed that turnover and nestedness processes contributed almost equally to the community distance of both viruses and host microbes, while the viral functional composition was dominated by turnover processes (Fig. 3e, f).

**Fig. 4:**
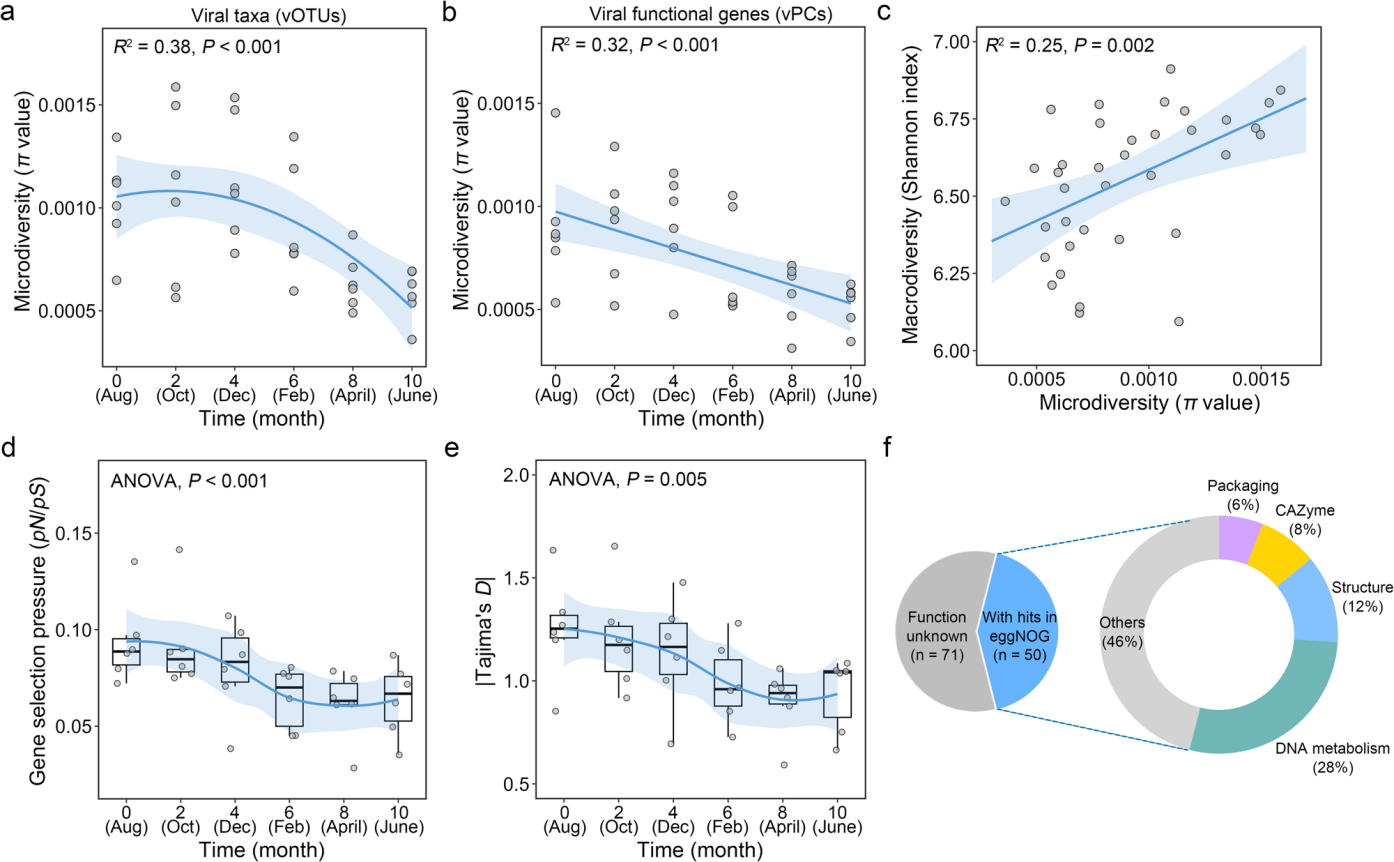
Temporal turnovers of viral micro-diversity and selection pressure. **a**, **b** The variations in the micro-diversity (*π* values) of viral taxa (vOTUs) and viral functional genes (vPCs) with time. **c** The relationships between viral macro-diversity (Shannon-Wiener index) and micro-diversity (*π* values). **d**, **e** The selection pressure of viral functional genes indicated by *pN*/*pS* and Tajima’s *D* values across different sampling time points (month). Boxes represented the interquartile range between the first and third quartiles and the median. Whiskers denoted the lowest and highest values within 1.5 times the range of the first and third quartiles. The significances among different sampling time points were determined by analysis of variance (ANOVA). **f** Functional classes of viral genes under positive selection (i.e., *pN*/*pS* > 1) in intertidal zones. The functional classes were annotated according to the eggNOG database. For **a**–**c**, the Pearson’s correlation coefficients and *P* values of the regressions were presented. All statistical tests were two-tailed.

In addition to macro-diversity patterns, further analyses were also conducted to explore the temporal dynamics of viral micro-diversity, which reflects the nucleotide-level variations of viral communities from the population genetics perspective. As a result, a similar temporal pattern to viral macro-diversity was observed for micro-diversity over the sampling months (Fig. 4a, b). Consequently, a significant positive association between viral micro-diversity and macro-diversity was observed (Fig. 4c). Higher selection pressure was observed in August to December than that in February to June (Fig. 4d, e). A total of 121 viral genes (~0.25%) were under positive selection (*pN*/*pS* > 1) in at least one sample (Fig. 4f). Among these, viral genes related to DNA metabolism and structure were the most abundant (Fig. 4f), suggesting that these nucleotide metabolic genes may have been undergone strong positive selection to adapt the changing environment. In addition, ~8% of viral genes under positive selection encoded carbohydrate-active enzymes (Fig. 4f), indicating that positive selection may have also favored viruses carrying carbohydrate metabolism genes.

### Environmental drivers of the temporal dynamics of viral communities

**Fig. 5:**
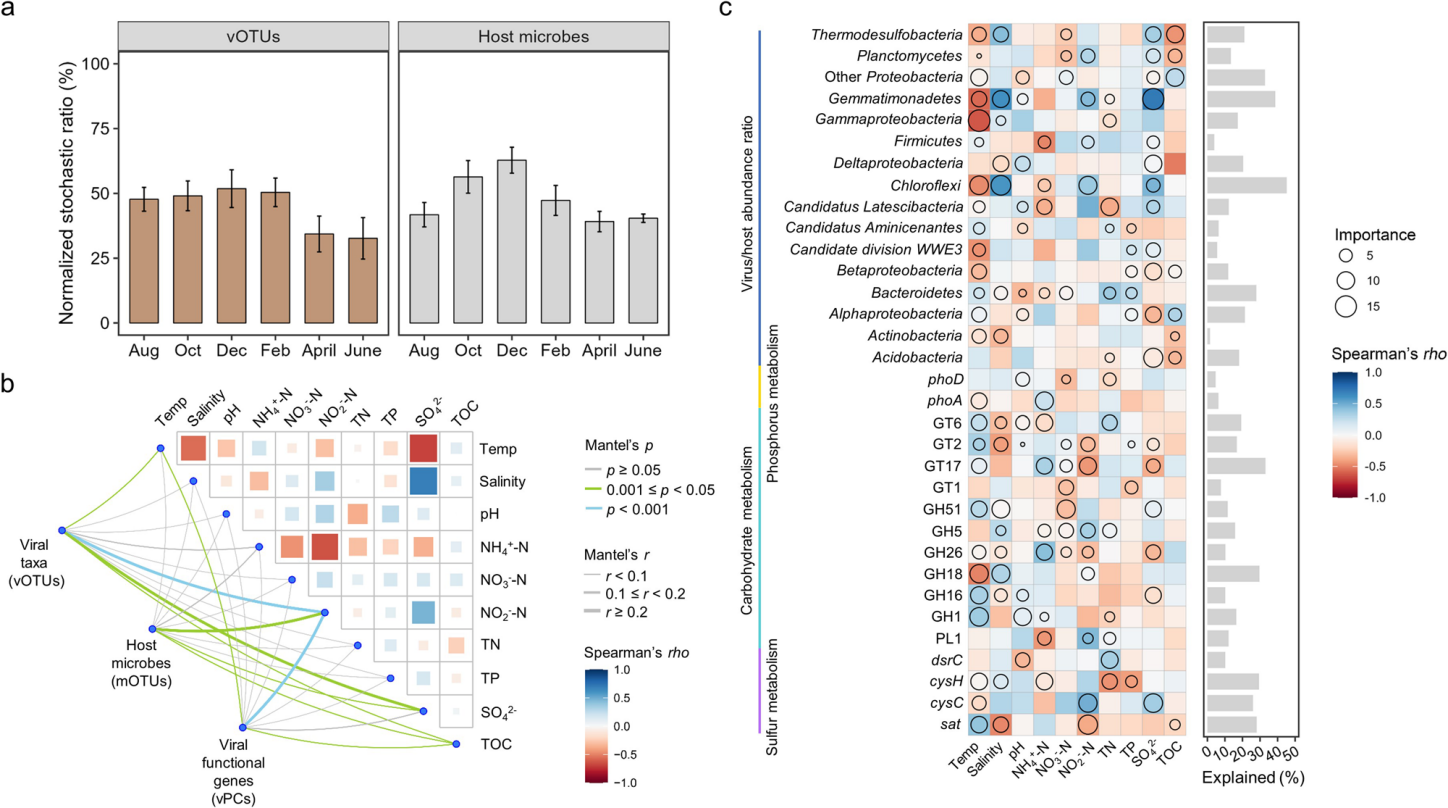
Environmental drivers of intertidal viruses and their host microbes. **a** Stochastic ratios of intertidal viral taxa (vOTUs) and their host microbes (mOTUs) along the sampling time. The normalized stochastic ratios were calculated based on 1000 null models. Stochastic ratios > 50% represented the dominance of stochastic processes. Data represented mean ± SEM. **b** Mantel tests showing the relationships between environmental factors and viral/microbial communities. The edge color and width represented Mantel’s *r* and *p*-value, res*p*ectively. The color gradient in the heatmap represented Spearman’s correlation coefficients between different environmental factors. **c** The importance of environmental factors in explaining the variations of viral auxiliary metabolic genes and virus–host relationships. The size of the circles represented the importance of environmental factors determined by random forest model analysis. The color gradient in heatmap represented the Spearman’s correlation coefficients. The bar plot represented the variations of viral auxiliary metabolic genes and virus–host relationships explained by all environmental factors included in random forest model analyses.

Both deterministic and stochastic processes, respectively grounded in ecological niche theory and neutral theory, have been shown to shape the compositional variations of microbial communities across various habitats in complementary ways^18^. Here, we first quantified the relative importance of deterministic and stochastic processes in controlling the intertidal viral biodiversity patterns. As a result, viral communities were similarly driven by deterministic and stochastic processes from August to February and dominated by deterministic processes in April and June (Fig. 5a). In contrast, host microbial communities exhibited stronger temporal variations of stochastic ratios than that of viral communities (Fig. 5a), indicating that the assembly of viral communities was more influenced by environmental factors than that of host microbial communities in intertidal zones.

A total of ten environmental factors were measured, most of which exhibited significant seasonal changes over time (Supplementary Fig. 8). Multiple statistical approaches were employed to explore the importance of environmental factors (deterministic processes) in driving the temporal dynamics of viral and host communities. First, mantel test suggested that variations in environmental factors were significantly correlated with the compositional variations of viral taxa (vOTUs) (Mantel’s *r* = 0.212, *P* < 0.001) and functional gene (vPCs) (Mantel’s *r* = 0.147, *P* < 0.01) (Fig. 5b). Multiple environmental factors, including temperature, nitrite nitrogen (NO_2_^−^–N), sulfate (SO_4_^2^^−^), and total organic carbon (TOC), were significantly associated with viral communities (Fig. 5b). Interestingly, most of these environmental factors exhibited distinct temporal trends divided by the winter season (December or February), similar to viral biodiversity patterns (Supplementary Fig. 8). Second, significant decay pattern with increasing environmental heterogeneity was observed for viral communities, but not for host microbial communities (Supplementary Fig. 9a). In addition, variation partitioning analyses suggested that the significantly correlated environmental factors explained a higher portion of the variations in viral communities (16.7%) than that in host microbial communities (10.9%) (Supplementary Fig. 9b). Such result demonstrated that environmental factors exerted stronger influence on the intertidal viral communities than host microbial communities. Third, random forest models were used to evaluate the relative importance of these significantly correlated environmental factors in driving viral biodiversity (Supplementary Fig. 9c). The results showed that SO_4_^2−^ and temperature were the major factors affecting viral β-diversity, while temperature and TOC were the major factors affecting viral α-diversity (Supplementary Fig. 9c).

In addition to community-level influences, strong associations were also observed between environmental factors and individual AMGs, as well as virus–host dynamics (Fig. 5c). Among these, temperature and salinity were the factors correlated with the relative abundances of most AMGs (Fig. 5c). Also, temperature affected the VHRs of almost all the microbial lineages. Notably, we observed that the VHRs of microbial lineages with highly explained variations by environmental factors, such as *Chloroflexi* and *Gemmatimonadetes*, exhibited strong fluctuations with time (Fig. 5c and Supplementary Table 8). Such results demonstrated that the fluctuations in virus–host relationships for specific lineages over time were largely driven by variations in environmental factors, especially the temperature.

## Discussion

A central goal in viral ecology is to resolve the patterns and ecological mechanisms that how the viral biodiversity is generated and maintained in complex natural ecosystems across space and through time^11^. Over the past years, the employment of multi-omics approaches has demonstrated that even the tiny viral particles also followed typical spatial scaling patterns (e.g., DDR) in complex ecosystems^12,13,21^. However, how viral communities change over time in complex natural ecosystems remains less studied. In this study, the viral biodiversity in a coastal intertidal zone was investigated bimonthly from both macro-diversity and micro-diversity angles, aiming to reveal the underlying ecological mechanisms driving the temporal turnover patterns of viral communities. The macro-diversity represents the inter-population diversity and is often described by α- and β-diversity, which has been largely explored in viromes across various ecosystems^12,17^. For both α- and β-diversity, strong and comparable temporal turnover patterns (TTR and TDR) were observed for viral communities and their host microbes. Comparatively, viruses were found with stronger TTR patterns than their host microbes, suggesting faster temporal taxa turnover of viruses^22^. In addition, temperature emerged as the most critical factor influencing intertidal viral communities, with winter serving as a pivotal point for the shifts in viral biodiversity. Consistently, multiple statistical analyses revealed that the variations in environmental factors exerted greater impacts on viral communities than on host microbial communities. These findings collectively underscored the rapid responses of intertidal viruses to seasonal changes.

Compared to viral taxa, viral functional genes were found with stronger temporal turnover in the intertidal zone. Mechanically, turnover rather than nestedness was the main driver for viral functional genes. This suggested that intertidal viruses carrying dissimilar functional genes (e.g., AMGs) were favored under different environmental conditions in different seasons. In addition, we also speculated that intertidal viromes may have frequently acted as agents mediating horizontal gene transfer processes^5,6^. In general, high selection pressure usually increases the instability and mutation rates of functional genes^28^. In this study, multiple viral genes were under positive selection pressure. Therefore, in addition to environmental selection, virus-mediated horizontal gene transfer may also contribute as an important reason for the high temporal turnover of viral functional genes.

Besides macro-diversity, the temporal patterns of viral micro-diversity were also investigated. Micro-diversity (i.e., intra-population diversity) is represented by nucleotide diversity and offers insights into population-level selection pressures^16,29^. For large organisms, the macro- and micro-diversity are often correlated across habitats sharing similar species pools^17,30^. However, a similar scenario is not observed for viral communities in the global ocean^17^. In this study, significant correlations and similar temporal patterns were observed for viral macro- and micro-diversity. This finding is consistent with a recent study of ocean viruses at the regional scale^16^. This suggested that the viral compositional heterogeneity at large spatial scales may have hampered the association between macro- and micro-diversity^15^.

Recently, a growing number of studies have focused on the viral mediations of biogeochemical cycles via encoding AMGs^14,31^. Multiple AMGs have been identified, such as the *dsrC* gene related to sulfate reduction^14^ and *amoC* gene related to nitrification^31^. In addition, virus-encoded CAZymes have also been recovered in environmental metagenomes and experimentally verified through cloning and expression^32^. However, limited knowledge is available regarding the stability of AMGs within viral genomes. In this study, AMGs related to carbohydrate, phosphorus, and sulfur metabolism pathways were identified in viral genomes. Phylogenetic analyses revealed the potential microbial hosts for these AMGs. Most of these AMGs were persistently detected in viral genomes over the sampling time, underscoring their importance for viral survival and metabolism. Strong associations were observed between these AMGs and multiple intertidal environmental factors, demonstrating that environmental variations may influence the relative abundances of viral AMGs. Strikingly, a considerable portion of viral functional genes under positive selection pressure were related to nucleotide metabolism processes, indicating that functionally important viral genes may be subject to a faster evolutionary rate than other genes as they adapt to host microbes^16^.

Owing to its ubiquity and high abundance, viruses are thought to be a major force in shaping the composition of microbial communities^2^. Here, the recovered intertidal viruses were observed to infect almost all microbial lineages. Previous work has demonstrated that the variations of virus–host relationships across space and through time may reflect the degree of responses to environmental factors^32–34^. Consistent with these findings, the VHRs of different host lineages in intertidal zones were associated with multiple environmental factors, especially temperature, suggesting that seasonal changes were important in driving virus–host relationships. Although covaried dynamic compositions were observed for viral and microbial communities, the overall virus–host networks and VHRs did not significantly vary through time at the whole community level, indicating relatively stable infection dynamics of intertidal viromes, as also observed for the human viromes^35^. Such stable virus–host interactions are expected to be beneficial for maintaining the stability of the intertidal ecosystem.

Insights were also gained for the life strategies of intertidal viromes. Two different viral predation models, including “Kill-the-Winner”^7^ and “Piggyback-the-Winner”^8^, represent the major life strategies of viruses in various ecosystems. The “Kill-the-Winner” model proposes that more viruses select lysis targeting the abundant host lineages, which can help regulate microbial communities by reducing the dominance of abundant lineages^7^. In contrast, the “Piggyback-the-Winner” model suggests that increasing microbial abundance leads to a lysis-lysogeny switch, resulting in decreased VHRs^8^. These two models have been observed in different habitats, representing different strategies of viral adaptation to the environment. Here, a counterbalanced viral life strategy was observed in the intertidal zone: lysis was adopted by viruses when host abundance was low, leading to a linear relationship between viruses and their host. As host abundance increases, viruses may adjust their lifestyles and infection strategy for specific lineages, leading to a decrease in VHRs when host abundance is high^13,36^. We speculated that the dynamic intertidal environmental conditions may be an important factor causing the changes in viral life strategies.

In conclusion, this study comprehensively investigated the temporal turnover patterns of intertidal viral community, host microbes, as well as infection dynamics in an intertidal wetland. Viral communities were found to follow strong and comparable temporal patterns as their host microbes. Viral functional genes were found with faster temporal turnovers than community composition. Multiple AMGs were stably present in the recovered intertidal viruses and significantly correlated with environmental factors. Virus–host interactions at the whole community level were relatively stable with time, but the VHRs of different host lineages varied. This study provided critical insights into the temporal turnovers of viromes in complex natural ecosystems, demonstrating the contribution of viruses to ecosystem stability and multifunction.

## Methods

### Experimental location and sample collection

The research sites were situated at Qingdao (36.46°N, 120.75°E), within the Shandong province of China. The intertidal sediments were collected in a time-series manner (every two months) at the research site spanning from August 2020 to June 2021. For each sampling time point, 15 different sedimental samples were collected, of which 6 were subjected to shotgun metagenome sequencing. For each sample, five sedimental cores were collected from 0–15 cm within 1 m^2^ and then homogenized as one sample.

### Measurement for environmental factors

A total of ten environmental factors were quantified, including temperature, salinity, pH, ammonia nitrogen (NH_4_^+^–N), nitrate nitrogen (NO_3_^−^–N), nitrite nitrogen (NO_2_^−^–N), total nitrogen (TN), total phosphorous (TP), sulfate (SO_4_^2−^), and total organic carbon (TOC). Among these, the temperature of sediments was measured in situ employing a mercury thermometer (−50 °C to 50 °C). The salinity of sediments was measured using a salinity meter (WS-31, Xudu, Beijing, China). The pH of sediments was determined with a pH meter (STARTER 300, OHAUS, Beijing, China). The TOC of sediments was measured using the TOC-L CPH meter (Shimadzu, Kyoto, Japan). Furthermore, the concentrations of NH_4_^+^–N, NO_3_^−^–N, NO_2_^−^–N, TN, TP, and SO_4_^2−^ were quantified through the utilization of spectrophotometry (Cytation5, BioTek, USA).

### DNA extraction and high-throughput sequencing

For the extraction of total DNA, 0.5 g of the homogenized sediment was used for each sample. The extraction process was executed using FastDNA SPIN kits designed for soil (MP Biochemicals, USA), following the precise guidelines laid out by the manufacturer. The total DNA was subsequently subjected to sequencing on the Illumina NovaSeq 6000 platform (paired-end, 2 × 150 bp, Inc., San Diego, CA, USA). For each sample, the sequence data amounts ranged from 20.3 Gb to 28.2 Gb, with an average data size of 22.4 Gb. The high-throughput sequencing procedure was conducted by NovoGene Co., Ltd. (Tianjin, China).

### De novo assembly

Raw reads of each intertidal sediment sample were input to Trimmomatic v0.39 (default parameters) to remove adapters and filter low-quality reads^37^. The clean reads of intertidal samples at each time point were co-assembled (i.e., assembly of multiple samples) using MEGAHIT v1.2.9 with k-mers of 29, 39, 59, 79, 99, 109, 127^38^.

### Identification and recovery of metagenomic viral contigs (mVCs)

First, co-assembled contigs ≥5 kb were extracted for viral identification. Virsorter2 v2.2.3 (score ≥ 0.7 and had at least one hallmark gene)^39^, DeepVirFinder v1.0 (score ≥ 0.7 and *p* < 0.05)^40^, and VIBRANT v1.2.1 (default parameters)^41^, were used to identify mVCs. Among these, mVCs identified by DeepVirFinder and VIBRANT were further input to CheckV v1.0.1^42^, and mVCs containing hallmark genes were retained. Notably, we also retained mVCs without hallmark genes but simultaneously identified by VirSorter2 (score ≥ 0.7), DeepVirFinder (score ≥ 0.7 and *p* < 0.05), and VIBRANT. All recovered mVCs were subsequently subjected to CheckV to exact proviral regions^42^. Only mVCs with length ≥10 kb or complete/high-quality genomes were retained for downstream analysis. The retained mVCs were de-replicated and clustered into vOTUs at 95% average nucleotide identity and 85% alignment fraction of shorter genomes^42^. Finally, the genome quality of each vOTU was assessed by CheckV.

### Taxonomic assignment and lifestyle prediction of viruses

Here, geNomad was used to assign taxonomic lineages to viruses following the latest ICTV taxonomy release^43^. Afterwards, a viral gene-sharing network was constructed to link mVCs at different time points using vConTACT2 v0.11.3^27^. Viral lifestyles were determined by identifying lysogenic signals and using deep-learning models. In brief, viral proteins were first input to eggNOG-mapper^44^ and VIBRANT^41^ to identify marker genes (integrase, recombinase, transposase, or repressor) to determine their lysogeny^14^. Viruses without marker genes were further input to PhaTYP^45^ to distinguish their lifestyles.

### Recovery and clustering of metagenome-assembled genomes (MAGs)

Here, three tools integrated in the metaWRAP v1.3.2 pipeline^46^, including MetaBAT v2.12.1^47^, MaxBin v2.2.6 (–universal)^48^, and CONCOCT v1.0.0^49^, were used to recover MAGs from co-assembled contigs (≥ 1.5 kb) from each time point. Subsequently, the metaWRAP module “Bin_refinement” was harnessed, applying criteria of over 50% completeness and less than 10% contamination to further streamline the retrieved MAGs into the final bin set^46^. The MAGs present within the final bin collection were clustered into mOTUs at 95% average nucleotide identity using dRep v3.4.0^50^. Finally, GTDB-tk v2.1.1^51^ based on the Genome Taxonomy Database R07-RS207 v2 was used to assign taxonomy to mOTUs.

### Host prediction of viruses

To optimize the assignment of host information to viruses, 3238 mVCs and 1462 MAGs were linked using multiple genomic features. Initially, viral genomes were aligned with MAGs, and the matches with hits ≥ 2000 bp and identity ≥70% identity were retained^15^. Next, the identification of tRNA genes within viral genomes was executed utilizing tRNAscan-SE v2.0.11^52^. These genes were subsequently run against MAGs with identity and coverage ≥ 95%. Meanwhile, the identification of CRISPR spacers within MAGs was identified^53^. The CRISPR spacers sequences were then run against mVCs with e-value ≤ 1e−5, identity ≥95%, and mismatch ≤1. Then, VirHostMatcher v1.0^54^ was employed to gauge the oligonucleotide frequency between mVCs and MAGs, and the matches with *d*_2_* values < 0.25 were retained. Finally, iPHoP v1.3.2^55^ was used to predict viral hosts with a confidence score of 90 (i.e., false discovery rate < 10%), and only the predicted hosts that matched MAGs obtained from this study were retained.

### Functional gene annotation of intertidal viruses

A total of 41,363 viral protein clusters (vPCs) were subjected to functional annotation by searching against eggNOG database v5.0 with an e-value cutoff of 1e−5 and a bit score of 50^44^. Auxiliary metabolic genes (AMGs) encoded by vOTUs were subsequently refined using the following two methods: (i) DRAM-v v1.4.6 pipeline^56^. vOTUs were input to Virsorter2 v2.2.3^39^ with ‘prep-for-dramv’ functions to screen contigs for performing DRAM-v annotation; (ii) VIBRANT v1.2.1 pipeline^41^. vOTUs were input to VIBRANT to identify AMGs and provide annotation information based on KEGG databases.

### Genomic analyses of intertidal viruses

Comparative genomic analyses of viruses were performed using clinker v0.0.23^57^. The functions of these viruses were annotated by screening against the NCBI-nr database using BLASTp (bit score > 50, and e-value < 1e−5). The homologs of viral *dsrC* and PL1 genes were determined by comparing the NCBI-nr database and MAGs in this study using BLASTp. The phylogenetic trees of *dsrC* and PL1 genes were constructed based on maximum likelihood algorithms in MEGA X with 500 bootstrap replicates^58^.

### Statistical analyses

Coverm v0.6.1 (https://github.com/wwood/CoverM) was used to calculate the coverage of the representative nucleotide sequences of vOTUs, mOTUs, and vPCs (parameters: identity ≥ 95%, coverage ≥ 90%, and ‘trimmed_mean’ mode). The coverage was further normalized according to the same read number (ten hundred million) and average read length (150 bp) for each sample. Other statistical analyses were performed based on multiple packages in R v4.2.0. The α-diversity (Richness and Shannon indices) and β-diversity (Bray–Curtis dissimilarity) were calculated using the vegan package^59^. The ‘beta.pair’ function of betapart package^60^ was used to calculate the Sørensen dissimilarity (*β*_sor_) and decomposed dissimilarity to turnover (*β*_sim_) and nestedness (*β*_sne_) processes. Micro-diversity (*π* value) and the selection pressures of viral genes (*pN*/*pS* and Tajima’s *D*) were calculated with MetaPop using default parameters^29^. The goodness of fit estimates between first and second-order polynomial models was compared using the corrected Akaike Information Criterion (AICc) calculated by nlme package^61^. Pearson/Spearman correlations were performed using the ‘rcorr’ function of Hmisc^62^. Environmental heterogeneity between different samples was calculated based on the Euclidean distance of normalized environmental factors. In the random forest model analyses, the relative importance (%lncMSE) of each environmental factor in driving specific viral functional traits and VHRs was estimated using randomForest package^63^. Specifically, the environmental factors added to each random forest model were screened by *P* value of %IncMSE calculated by rfPermute package and the explained variation (*R*^2^) calculated by A3 package^64^, until the random forest model was determined to be significant (*P* < 0.05). In addition, the A3 package^64^ was used to further evaluate the total explained variation of each random forest model. To disentangle the relative importance of deterministic and stochastic processes in underlying community assembly, a total of 1000 null models were analyzed using the NST package^65^. The stochastic ratios were calculated for each time point using taxonomic metrics based on the Bray–Curtis distance.

### Limitations of the employed methodology

Different approaches are available for viral ecology studies in complex environments, including metavirome and metagenome sequencing^10^. In this study, shotgun metagenome sequencing was employed to recover the genomic information for both viruses and host microbes, based on which comprehensive investigations were carried out for the intertidal viral communities. Comparatively, fewer viruses are usually recovered by metagenomes than metaviromes due to the large volume of non-viral sequences in metagenomes^66^, though some exceptions have also been observed^67^. However, metagenome sequencing holds the advantage of simultaneously targeting viruses and microbes, enabling virus–host/microbe analyses within the same dataset and under the same sequencing depth, avoiding complex data analysis issues from different sequencing batches and datasets^10^. Recently, concerns have been raised for VHR analyses, recommending the use of absolute abundance technologies^68^. Although absolute abundance is desired in microbial ecology studies^69^, absolute quantification of viruses based on flow cytometry/fluorescence microscopy can only provide limited information, lacking the taxonomy of both viruses and their hosts, not accounting for the high false positives for soil/sediment samples^70^. Notably, recent studies have also demonstrated promising virus–host relationship analyses using shotgun metagenomes^70,71^. In addition, as also observed in this study, the ratios of both taxonomic assignment and functional annotation for the recovered viral genomes were relatively low, mainly due to the insufficient coverage of viral sequences in public databases^10^. Despite of the potential limitations, metagenomic sequencing provides valuable insights into the ecological patterns of viruses, given the current constraints of available technical approaches.

### Supplementary information

Supplementary Information

Supplementary Table

## Data Availability

Raw reads of mudflat intertidal metagenomes generated in this study have been deposited in the NCBI Sequence Read Archive (SRA) database under project ID PRJNA957716 and PRJNA1029225. Detailed information on the shotgun metagenomes is listed in Supplementary Table 1. The sequences of vOTUs, MAGs, and vPCs generated from this study are available at 10.5281/zenodo.10012334.

## Code availability

R codes used for figure generation and statistical analyses in this study are publicly available on the GitHub repository.

## References

[1] Wasik BR, Turner PE (2013). On the biological success of viruses. Annu. Rev. Microbiol..

[2] Suttle CA (2007). Marine viruses—major players in the global ecosystem. Nat. Rev. Microbiol..

[3] Zimmerman AE (2020). Metabolic and biogeochemical consequences of viral infection in aquatic ecosystems. Nat. Rev. Microbiol..

[4] Albright MB (2022). Experimental evidence for the impact of soil viruses on carbon cycling during surface plant litter decomposition. ISME Commun..

[5] Howard-Varona C, Hargreaves KR, Abedon ST, Sullivan MB (2017). Lysogeny in nature: mechanisms, impact and ecology of temperate phages. ISME J..

[6] Correa AM (2021). Revisiting the rules of life for viruses of microorganisms. Nat. Rev. Microbiol..

[7] Winter C, Bouvier T, Weinbauer MG, Thingstad TF (2010). Trade-offs between competition and defense specialists among unicellular planktonic organisms: the “killing the winner” hypothesis revisited. Microbiol. Mol. Biol. Rev..

[8] Knowles B (2016). Lytic to temperate switching of viral communities. Nature.

[9] Roux S, Hallam SJ, Woyke T, Sullivan MB (2015). Viral dark matter and virus–host interactions resolved from publicly available microbial genomes. elife.

[10] Kieft K, Anantharaman K (2022). Virus genomics: what is being overlooked?. Curr. Opin. Virol..

[11] Roux S, Emerson JB (2022). Diversity in the soil virosphere: to infinity and beyond?. Trends Microbiol..

[12] Gao S (2022). Patterns and ecological drivers of viral communities in acid mine drainage sediments across Southern China. Nat. Commun..

[13] Ma B (2024). Biogeographic patterns and drivers of soil viromes. Nat. Ecol. Evolut..

[14] Kieft K (2021). Ecology of inorganic sulfur auxiliary metabolism in widespread bacteriophages. Nat. Commun..

[15] Roux S (2016). Ecogenomics and potential biogeochemical impacts of globally abundant ocean viruses. Nature.

[16] Zhong Z-P (2023). Lower viral evolutionary pressure under stable versus fluctuating conditions in subzero Arctic brines. Microbiome.

[17] Gregory AC (2019). Marine DNA viral macro-and microdiversity from pole to pole. Cell.

[18] Zhou, J. & Ning, D. Stochastic community assembly: does it matter in microbial ecology? *Microbiol. Mol. Biol. Rev*. 10.1128/mmbr.00002-17 (2017). 10.1128/MMBR.00002-17 PMC5706748 29021219

[19] Gaston KJ (2000). Global patterns in biodiversity. Nature.

[20] Liang Y (2015). Long-term soil transplant simulating climate change with latitude significantly alters microbial temporal turnover. ISME J..

[21] Fan X (2023). Global diversity and biogeography of DNA viral communities in activated sludge systems. Microbiome.

[22] Van Der Gast CJ, Ager D, Lilley AK (2008). Temporal scaling of bacterial taxa is influenced by both stochastic and deterministic ecological factors. Environ. Microbiol..

[23] Guo X (2018). Climate warming leads to divergent succession of grassland microbial communities. Nat. Clim. Change.

[24] Murray NJ (2019). The global distribution and trajectory of tidal flats. Nature.

[25] Wang F (2021). Global blue carbon accumulation in tidal wetlands increases with climate change. Natl Sci. Rev..

[26] Konstantinidis KT, Tiedje JM (2005). Genomic insights that advance the species definition for prokaryotes. Proc. Natl Acad. Sci. USA.

[27] Bin Jang H (2019). Taxonomic assignment of uncultivated prokaryotic virus genomes is enabled by gene-sharing networks. Nat. Biotechnol..

[28] Liao MJ, Din MO, Tsimring L, Hasty J (2019). Rock-paper-scissors: engineered population dynamics increase genetic stability. Science.

[29] Gregory AC (2022). MetaPop: a pipeline for macro-and microdiversity analyses and visualization of microbial and viral metagenome-derived populations. Microbiome.

[30] Vellend M, Geber MA (2005). Connections between species diversity and genetic diversity. Ecol. Lett..

[31] Gazitúa MC (2021). Potential virus-mediated nitrogen cycling in oxygen-depleted oceanic waters. ISME J..

[32] Emerson JB (2018). Host-linked soil viral ecology along a permafrost thaw gradient. Nat. Microbiol..

[33] Ji M (2023). Tundra soil viruses mediate responses of microbial communities to climate warming. Mbio.

[34] Coclet C (2023). Virus diversity and activity is driven by snowmelt and host dynamics in a high-altitude watershed soil ecosystem. Microbiome.

[35] Liang G, Bushman FD (2021). The human virome: assembly, composition and host interactions. Nat. Rev. Microbiol..

[36] Wigington CH (2016). Re-examination of the relationship between marine virus and microbial cell abundances. Nat. Microbiol..

[37] Bolger AM, Lohse M, Usadel B (2014). Trimmomatic: a flexible trimmer for Illumina sequence data. Bioinformatics.

[38] Li D, Liu C-M, Luo R, Sadakane K, Lam T-W (2015). MEGAHIT: an ultra-fast single-node solution for large and complex metagenomics assembly via succinct de Bruijn graph. Bioinformatics.

[39] Guo J (2021). VirSorter2: a multi-classifier, expert-guided approach to detect diverse DNA and RNA viruses. Microbiome.

[40] Ren J (2020). Identifying viruses from metagenomic data using deep learning. Quant. Biol..

[41] Kieft K, Zhou Z, Anantharaman K (2020). VIBRANT: automated recovery, annotation and curation of microbial viruses, and evaluation of viral community function from genomic sequences. Microbiome.

[42] Nayfach S (2021). CheckV assesses the quality and completeness of metagenome-assembled viral genomes. Nat. Biotechnol..

[43] Camargo AP (2023). Identification of mobile genetic elements with geNomad. Nat. Biotechnol..

[44] Cantalapiedra CP, Hernández-Plaza A, Letunic I, Bork P, Huerta-Cepas J (2021). eggNOG-mapper v2: functional annotation, orthology assignments, and domain prediction at the metagenomic scale. Mol. Biol. Evolut..

[45] Shang J, Tang X, Sun Y (2023). PhaTYP: predicting the lifestyle for bacteriophages using BERT. Brief. Bioinforma..

[46] Uritskiy GV, DiRuggiero J, Taylor J (2018). MetaWRAP—a flexible pipeline for genome-resolved metagenomic data analysis. Microbiome.

[47] Kang DD (2019). MetaBAT 2: an adaptive binning algorithm for robust and efficient genome reconstruction from metagenome assemblies. PeerJ.

[48] Wu Y-W, Simmons BA, Singer SW (2016). MaxBin 2.0: an automated binning algorithm to recover genomes from multiple metagenomic datasets. Bioinformatics.

[49] Alneberg J (2014). Binning metagenomic contigs by coverage and composition. Nat. Methods.

[50] Olm MR, Brown CT, Brooks B, Banfield JF (2017). dRep: a tool for fast and accurate genomic comparisons that enables improved genome recovery from metagenomes through de-replication. ISME J..

[51] Chaumeil P-A, Mussig AJ, Hugenholtz P, Parks DH (2022). GTDB-Tk v2: memory friendly classification with the genome taxonomy database. Bioinformatics.

[52] Chan PP, Lin BY, Mak AJ, Lowe TM (2021). tRNAscan-SE 2.0: improved detection and functional classification of transfer RNA genes. Nucleic Acids Res..

[53] Rho M, Wu Y-W, Tang H, Doak TG, Ye Y (2012). Diverse CRISPRs evolving in human microbiomes. PLoS Genet..

[54] Ahlgren NA, Ren J, Lu YY, Fuhrman JA, Sun F (2017). Alignment-free oligonucleotide frequency dissimilarity measure improves prediction of hosts from metagenomically-derived viral sequences. Nucleic Acids Res..

[55] Roux S (2023). iPHoP: an integrated machine learning framework to maximize host prediction for metagenome-derived viruses of archaea and bacteria. PLoS Biol..

[56] Shaffer M (2020). DRAM for distilling microbial metabolism to automate the curation of microbiome function. Nucleic Acids Res..

[57] Gilchrist CL, Chooi Y-H (2021). Clinker & clustermap. js: automatic generation of gene cluster comparison figures. Bioinformatics.

[58] Kumar S, Stecher G, Li M, Knyaz C, Tamura K (2018). MEGA X: molecular evolutionary genetics analysis across computing platforms. Mol. Biol. Evolut..

[59] Oksanen J (2013). Package ‘vegan’. Community Ecology Package Version.

[60] Baselga A, Orme CDL (2012). betapart: an R package for the study of beta diversity. Methods Ecol. Evolut..

[61] Pinheiro J, Bates D, DebRoy S, Sarkar D (2012). Nonlinear mixed-effects models. R Package Version.

[62] Harrell FE, Harrell MFE (2019). Package ‘hmisc’. CRAN2018..

[63] Liaw A, Wiener M (2002). Classification and regression by randomForest. R. N..

[64] Fortmann-Roe S (2015). Consistent and clear reporting of results from diverse modeling techniques: the A3 method. J. Stat. Softw..

[65] Ning D, Deng Y, Tiedje JM, Zhou J (2019). A general framework for quantitatively assessing ecological stochasticity. Proc. Natl Acad. Sci. USA.

[66] Santos-Medellin C (2021). Viromes outperform total metagenomes in revealing the spatiotemporal patterns of agricultural soil viral communities. ISME J..

[67] Bi L, He J-Z, Hu H-W (2024). Total metagenomes outperform viromes in recovering viral diversity from Sulfuric soils. ISME Commun..

[68] Alrasheed H, Jin R, Weitz JS (2019). Caution in inferring viral strategies from abundance correlations in marine metagenomes. Nat. Commun..

[69] Maghini DG (2024). Quantifying bias introduced by sample collection in relative and absolute microbiome measurements. Nat. Biotechnol..

[70] López-García P (2023). Metagenome-derived virus-microbe ratios across ecosystems. ISME J..

[71] Roux S, Brum JR (2023). Counting dots or counting reads? Complementary approaches to estimate virus-to-microbe ratios. ISME J..

