# Supplementary material for: Temporal turnover of viral biodiversity and functional potential in intertidal wetlands
Source: NPJ Biofilms Microbiomes. 2024 Jun 19;10:48. doi: 10.1038/s41522-024-00522-8 (PMC11186824; doi:10.1038/s41522-024-00522-8)
Supplement: Supplementary file 1 — Supplementary Information [file 41522_2024_522_MOESM1_ESM.pdf]

Supporting Information for

Temporal turnover of viral biodiversity and functional potential in intertidal wetlands

Mengzhi Ji, Yan Li, Jiayin Zhou, Wen Song, Yuqi Zhou, Kai Ma, Mengqi Wang, Xia Liu, Yueyue Li, Xiaofan Gong, Qichao Tu

Corresponding author: Qichao Tu

Email: tuqichao@sdu.edu.cn

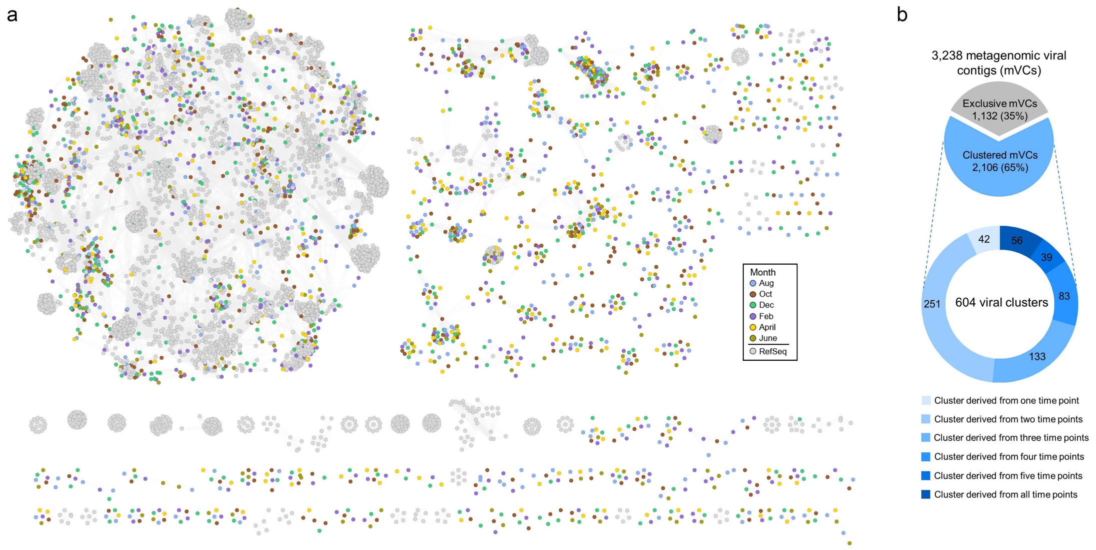

Supplementary Fig. 1. Linkages of intertidal viruses across different sampling time points. a The network was generated by vConTACT2 using intertidal viruses from different time points. The nodes (circle) represented different viral genomes and the edges represented shared protein cluster content. Different colors represented the sampling time point that the viral genomes were recovered. **b** The pie chart at the top represented the number of metagenomic viral contigs (mVCs) that were clustered and excluded in the network. The pie chart at the bottom represented the number of viral clusters aggregated by the mVCs from different time points.

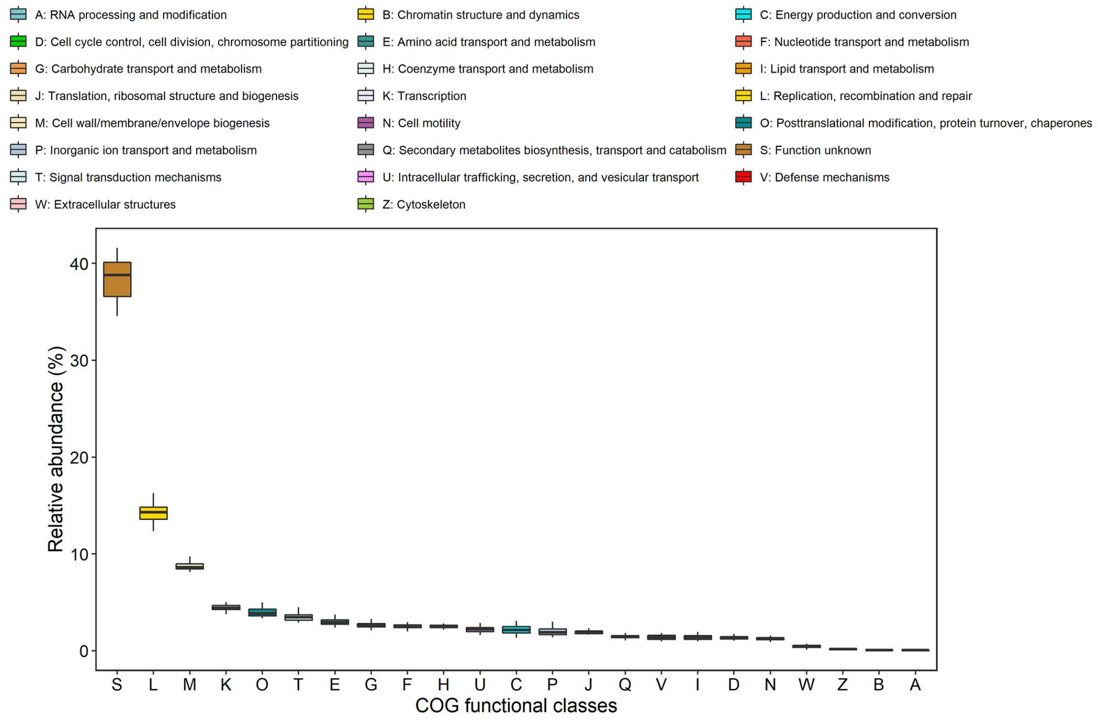

Supplementary Fig. 2. The relative abundances of viral functional genes categorized by different COG classes. Boxes represented the interquartile range between the first and third quartiles and the median (internal line). Whiskers denoted the lowest and highest values within 1.5 times the range of the first and third quartiles, respectively.

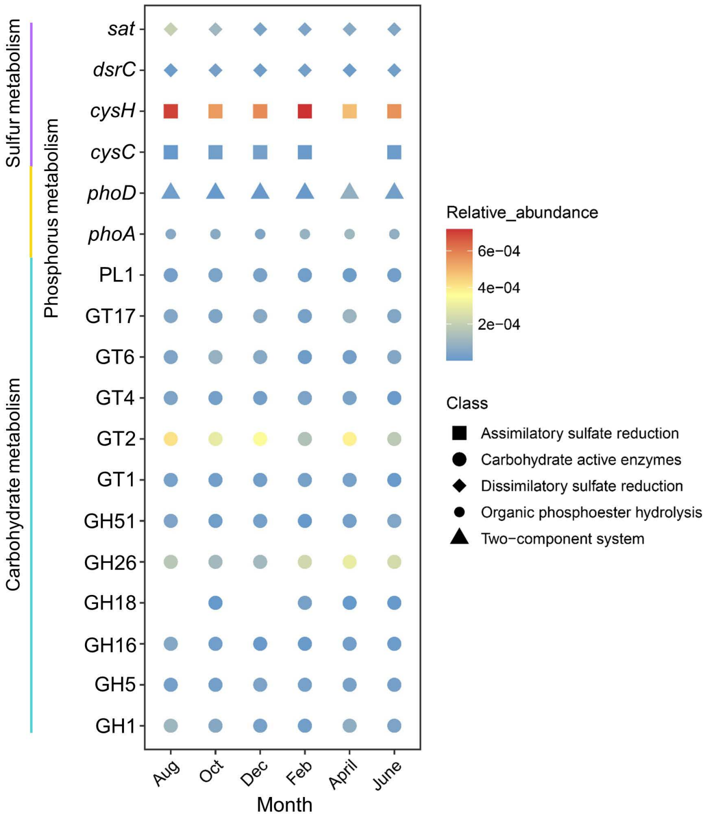

Supplementary Fig. 3. The relative abundances of viral auxiliary metabolic genes (vAMGs) related to carbohydrate, phosphorus, and sulfur metabolism along the sampling time points. The color intensity represented the relative abundances of vAMGs (abundances of vAMGs/total abundances of viral functional genes). Different shapes represented different vAMG classes.

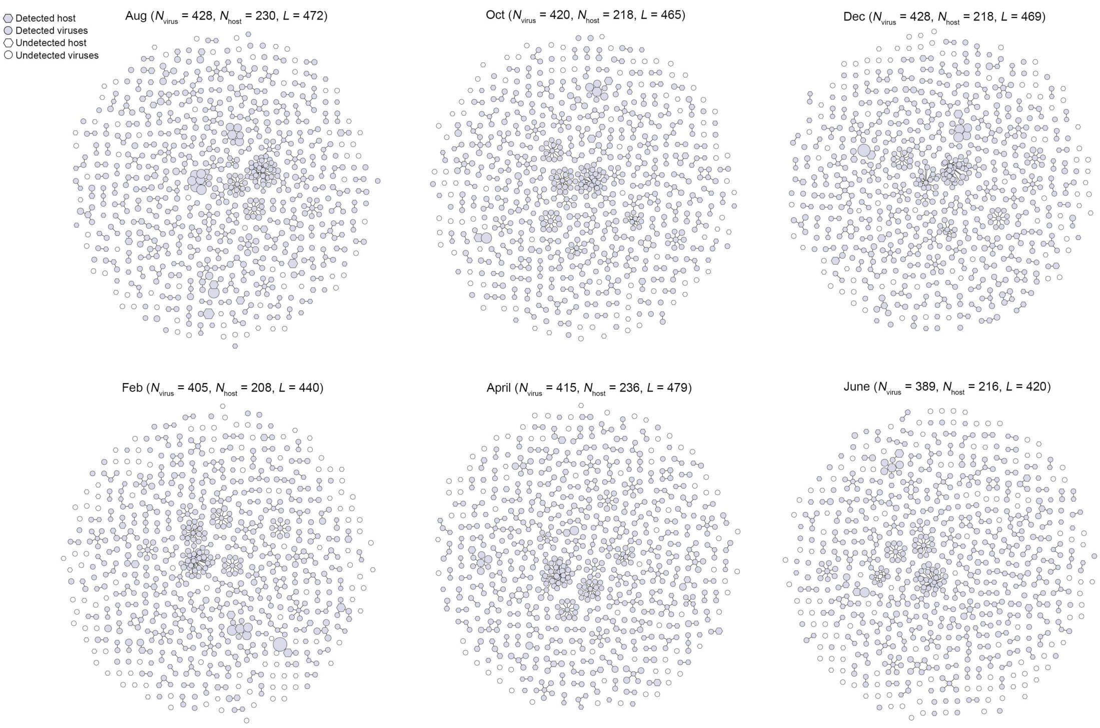

Supplementary Fig. 4. Virus-host interaction networks at different time points. The networks were constructed based on the virus-host pairs of 478 vOTUs and 249 host mOTUs. Only vOTUs/mOTUs with average read coverage (normalized abundance) > 1 at one of the time points were considered to be detected and colored by purple. Viruses (circles) were connected to their microbial hosts (hexagons) by edges if they were both detected at the same time point. *L* represented the number of virus-host pairs and *N* represented the number of detected viruses or host microbes at each time point. The size of nodes represented their normalized abundance. Undetected viruses or host microbes were shown in white with minimum size for visualization purposes.

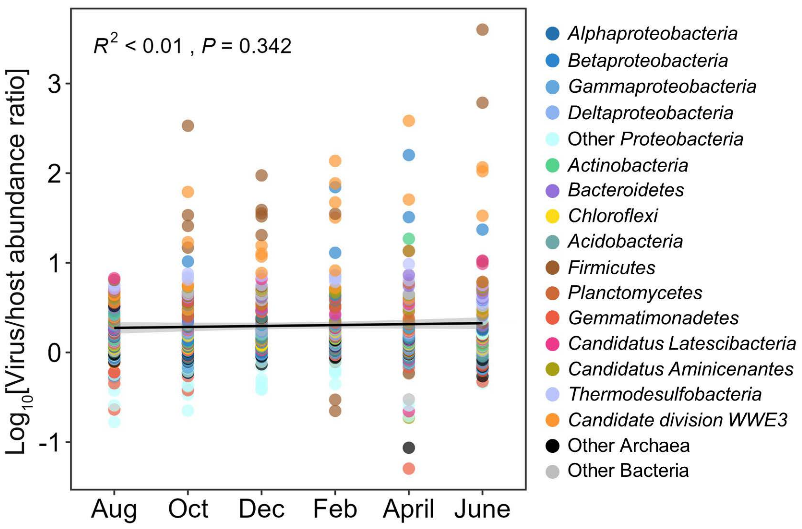

Supplementary Fig. 5. Virus/host abundance ratios (VHRs) along the sampling months. Different colors represented different microbial host lineages. Each point represented the VHR of each microbial lineage in each sample. The regression line represented the overall variations in the VHRs of the whole community with sampling time. The Pearson's correlation coefficient and *P* value of the regression were presented.

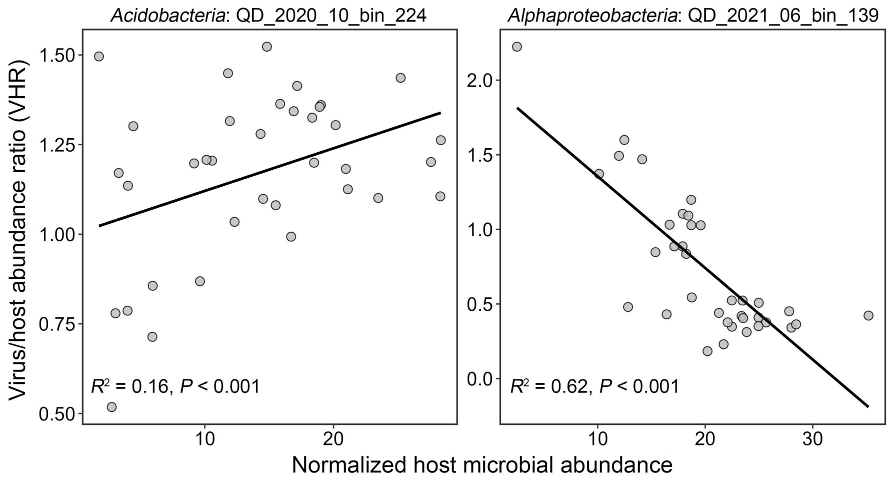

Supplementary Fig. 6. The relationships between normalized host abundances and their virus/host abundance ratios (VHRs) of virus-host pairs. The regressions represented the overall variations in the VHRs with host abundances. These virus-host pairs were detected in all samples, making them as the suitable candidates for analyses. The Pearson's correlation coefficients and *P* values of the regressions were presented.

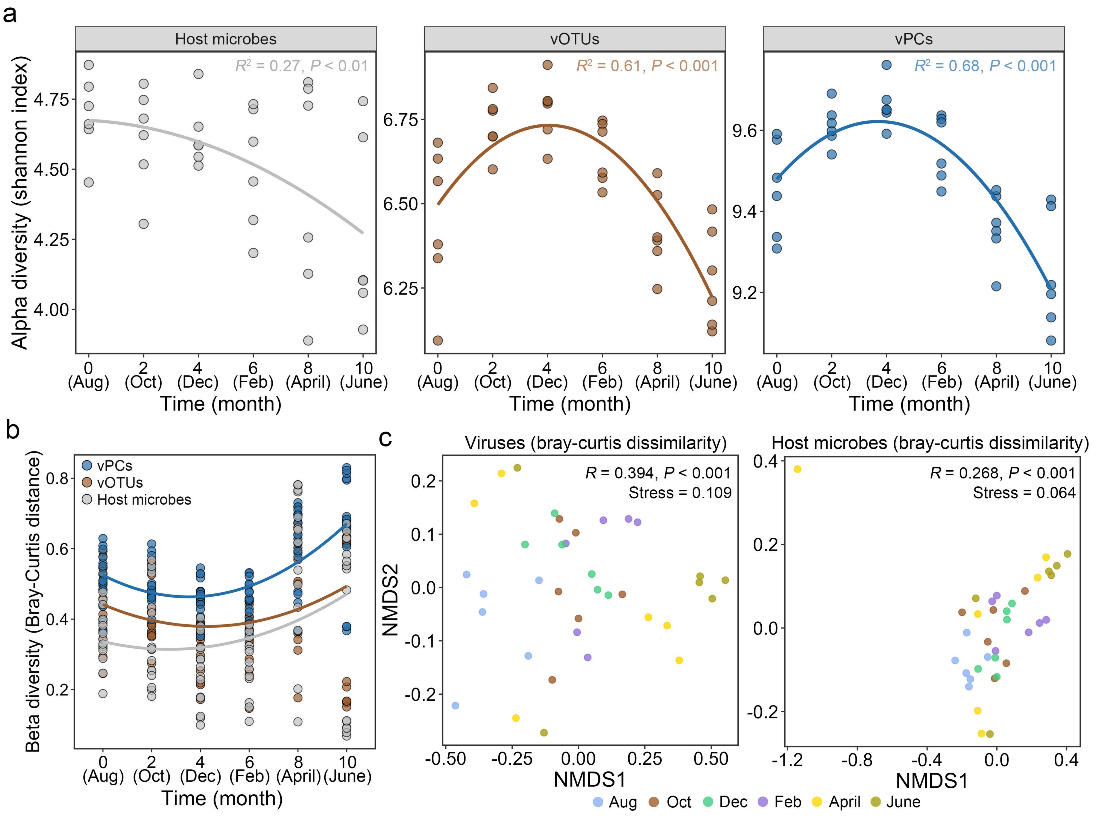

Supplementary Fig. 7. The α- and β-diversity of viral taxa (vOTUs), viral functional genes (vPCs), and host microbes (mOTUs). a The variations in the α-diversity (shannon index) of viral taxa, viral functional genes, and host microbes with time. The Pearson's correlation coefficients and *P* values of the regressions were presented. **b** The variations in the community distance of viral taxa, viral functional genes, and host microbes with time. Community distance was measured by Bray-Curtis dissimilarity. **c** Nonmetric multidimensional scaling analyses of viral and host microbial communities based on Bray-Curtis dissimilarity. The significance among different sampling time points was determined by analysis of similarity (ANOSIM) with 1000 permutations.

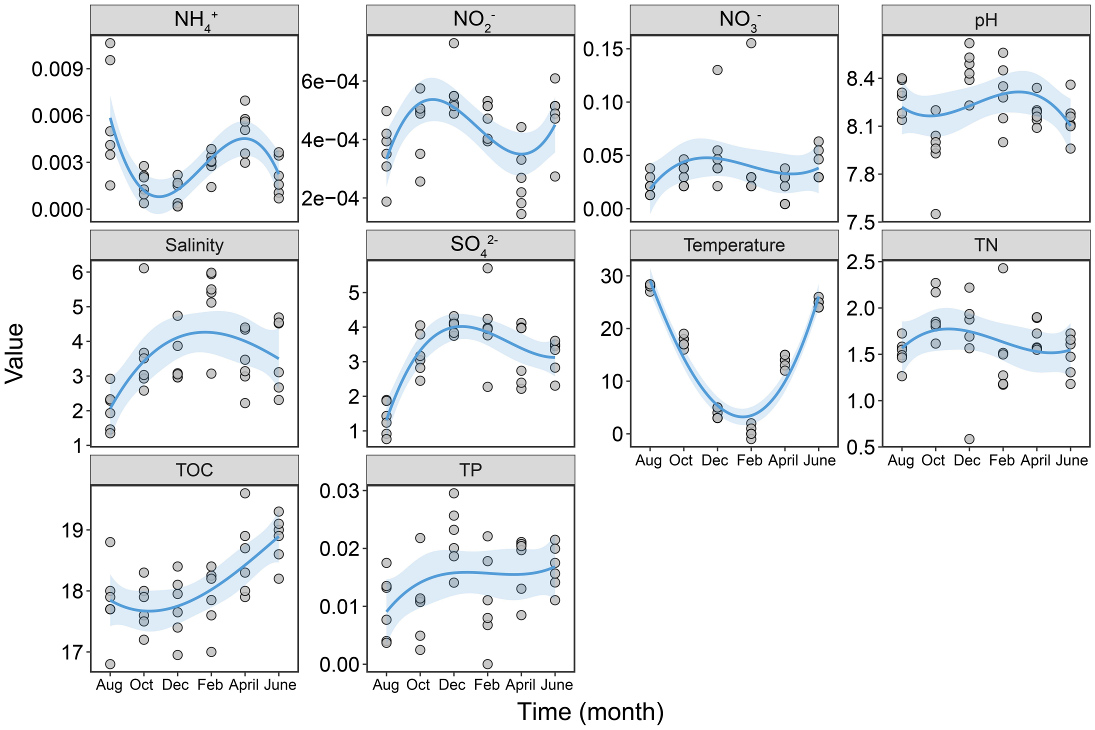

Supplementary Fig. 8. The variations of environmental factors with time. Regression models were employed to illustrate the relationships between environmental factors and sampling time (month). The fitted regression models were determined by the bs function in the splines R package.

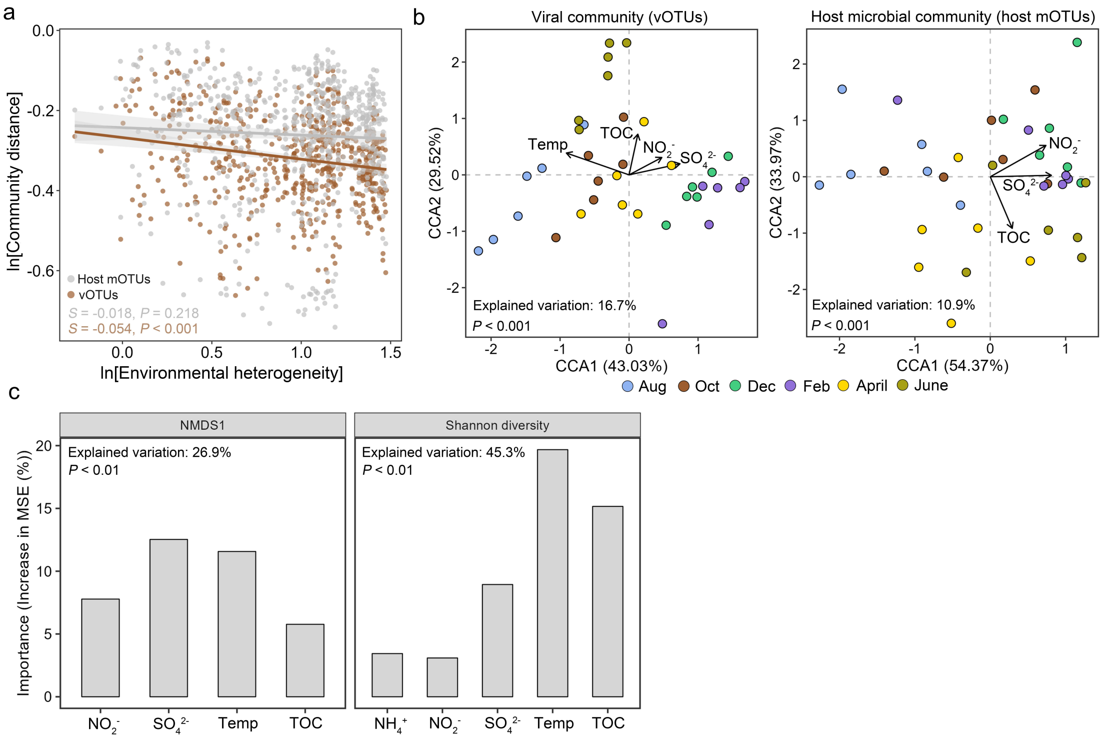

Supplementary Fig. 9. The relationships between viral/host community compositions and environmental factors. a The relationships between environmental heterogeneity and community distance. Environmental heterogeneity was calculated based on Euclidean distance, and the community distance was calculated based on Bray-Curtis dissimilarity. The Pearson's correlation coefficients and *P* values of the regressions were presented. **b** Canonical correspondence analysis (CCA) of viral taxa (vOTUs) and host microbes (mOTUs) and environmental factors. Only the environmental factors that exhibited significant correlations with viral or host microbial community compositions, as determined by the Mantel test, were utilized for CCA. Variation partitioning analysis (VPA) was used to estimate the contributions of environmental factors in explaining the compositional variations of viral and host microbial communities (adjust R^2). Permutation test was used to determine the significance. **c** The importance of key environmental factors in explaining the α-

(Shannon-Wiener index) and β-diversity (NMDS1) of viruses. Only the environmental factors that exhibited significantly correlations with viral α- or β-diversity were included in the random forest models. The increase in mean squared error (MSE) calculated by randomForest reflected the relative importance of each environmental factor in explaining viral α- or β-diversity. The significances of random forest models were determined by the A3 package in R.
